# Retraction Note: Hypericin-mediated photodynamic therapy induces apoptosis of myoloma SP2/0 cells depended on caspase activity in vitro

**DOI:** 10.1186/s12935-015-0199-8

**Published:** 2015-06-24

**Authors:** Junping Zhang, Linxiang Shao, Chunlin Wu, Hongfei Lu, Ruian Xu

**Affiliations:** 1 Engineering Research Center of Molecular Medicine, Ministry of Education, China and School of Medicine, Huaqiao University, 269 Chenghua North Road, 361021 Quanzhou, Fujian Province China; 2 Department of Bioscience, College of Chemistry and Life Science, Zhejiang Normal University, 321004 Jinhua, China; 3 Department of Pathology, Second Affiliated Hospital of Fujian Medical University, 36200 Quanzhou, China

## Retraction

The Publisher and Editor regretfully retract this article [1] because the peer-review process was inappropriately influenced and compromised. As a result, the scientific integrity of the article cannot be guaranteed. A systematic and detailed investigation suggests that a third party was involved in supplying fabricated details of potential peer reviewers for a large number of manuscripts submitted to different journals. In accordance with recommendations from [COPE] we have retracted all affected published articles, including this one. It was not possible to determine beyond doubt that the authors of this particular article were aware of any third party attempts to manipulate peer review of their manuscript.
